# Metabolic syndrome in hypertensive and non‐hypertensive subjects

**DOI:** 10.1002/hsr2.454

**Published:** 2021-12-14

**Authors:** Roshali Senarathne, Usha Hettiaratchi, Nirodha Dissanayake, Riyaza Hafiz, Sumara Zaleem, Lohini Athiththan

**Affiliations:** ^1^ Faculty of Allied Health Sciences University of Sri Jayewardenepura Nugegoda Colombo Sri Lanka; ^2^ Department of Biochemistry, Faculty of Medical Sciences University of Sri Jayewardenepura Nugegoda Colombo Sri Lanka

**Keywords:** hypertension, metabolic syndrome, waist circumference

## Abstract

**Background and aims:**

Hypertension is a major risk factor of cardiovascular diseases (CVDs), which attributes to one‐third of all deaths worldwide. It is also considered as a key feature of metabolic syndrome (MetS). The aim of the present study was to compare the presence of characteristic features of MetS in hypertensive and non‐hypertensive males and females and find out the percentages of MetS in hypertensive and non‐hypertensive adults.

**Methods:**

This was a cross‐sectional study, involving 120 participants that included 60 hypertensives and 60 non‐hypertensives (35‐55 years). Data were obtained through an interviewer‐administered questionnaire. Fasting blood sugar (FBS) and lipid parameters [triglyceride‐(TG), high density lipoprotein (HDL)] were analyzed, and waist circumference (WC) was measured. Percentages of MetS among hypertensive and non‐hypertensive groups were determined according to both modified Adult Treatment Panel III (ATP III) and new International Diabetes Federation (IDF) criteria. Results were analyzed using SPSS version 21.

**Results:**

Among the characteristic features of MetS, mean FBS and WC were significantly higher in hypertensive males compared with non‐hypertensive males (*P* < .001 and *P* = .002 respectively), while mean value of TG was significantly higher (*P* = .005) in hypertensive females compared with non‐hypertensive females. Further, the percentage of subjects in the total hypertensive group with increased FBS and increased WC was significantly higher than the non‐ hypertensive group. The percentage of subjects with MetS was significantly (*P* < .001) higher in hypertensive group (68%) compared with non‐hypertensive group (20%) according to modified ATP III criteria. When compared with new IDF criteria, it was 63% and 20%, respectively.

**Conclusion:**

The percentage of subjects with increased FBS, WC, and MetS was significantly higher in the hypertensive group compared with non‐hypertensives group. These findings emphasize the urgent need to develop national strategies for early detection, and to take preventive measures to make people aware of the impact of metabolic syndrome.

## 1 INTRODUCTION

Hypertension is a major health risk factor attributing to increase global mortality both in developed as well as in developing countries with relatively higher prevalence in developing countries.^1^ A systematic review, which was done using the published studies from the year 2000 to 2013 in eight South Asian countries, has observed the prevalence of hypertension as follows: Bangladesh: 17.9%; Bhutan: 23.9%; India: 31.4%; Maldives: 31.5%; Nepal: 33.8%; Pakistan: 25%; and Sri Lanka: 20.9%.^2^ Another study by Katulanda et al, in the Sri Lankan population (2009), has observed the prevalence of hypertension in total, males, and females as 23.7%, 23.4%, and 23.8 %, respectively.^3^ Hypertension is not only a major risk factor of CVD that attributes to about one‐third of all deaths worldwide but also considered as a key feature of MetS. Abdominal obesity and insulin resistance have been identified as the predominant underlying risk factors for MetS, while atherogenic dyslipidemia and hyperglycemia are considered as other common features of MetS.^4^, ^5^

Worldwide prevalence of MetS has increased significantly over the years. Studies have shown approximately 20% to 25% of the world's adult population have MetS and they are prone to have a threefold greater risk for CVD morbidity and fivefold greater risk of developing type 2 diabetes mellitus (T2DM).^6^ A systematic review in 2016 has summarized the mean prevalence of metabolic syndrome in South Asian countries as 14.0% (WHO), 26.1% (ATPIII), and 29.8% (IDF).^7^ In addition, the prevalence of MetS among Sri Lankan adults is increasing at an alarming rate, where one‐fourth of the Sri Lankan population were identified as MetS during the year 2005 to 2006.^8^ The prevalence of MetS in a Sri Lankan urban population in 2014 was 46.2% with 28.6% among males and 61.4% among females.^9^

As hypertension is a key feature of MetS and could be measured easily, this could be used as an important measure to predict the development of MetS. As no studies have been carried out to identify MetS among hypertensives in Sri Lanka, this study would provide an insight to compare the characteristic features of MetS in hypertensive and non‐hypertensive males and females and to find out the percentage of MetS in hypertensive and non‐hypertensive groups. Many studies have pointed out the importance of preventing MetS in order to reduce morbidity and mortality due to CVD and T2DM.^10^, ^11^ If MetS could be predicted earlier, when individuals have lesser number of characteristics, preventive measures such as increased physical activity and dietary control and also medications when necessary can be administered.^12^

## 2 METHODS

### 2.1 Study design and participants

A cross‐sectional study was carried out at the Family Practice Centre of University of Sri Jayewardenepura, Nugegoda, Sri Lanka during September 2015‐January 2016. The study was approved by the Ethics review committee of Faculty of Medical Sciences, University of Sri Jayewardenepura (reference numbers‐ MLS 06/2015, B Pharm 05/2015). This study involved 120 participants between the age range of 35 to 55 years, including 60 hypertensives and 60 non‐hypertensives.

Informed written consent was obtained from all the participants before the study. Hypertensive adults (diagnosed as hypertension >140/90 mmHg and/or on hypertensive drugs) were included in the “Test” group and non‐hypertensive adults who were not diagnosed for hypertension and with normal blood pressure <120/80 mmHg, were included in “Control” group. Subjects who were pregnant, having severe diseases, physical impairments, and who did not like to participate in the study were excluded. The female and male distribution in each hypertensive and non‐hypertensive group were similar (n = 30). Socioeconomic data, lifestyle information, and medical information were obtained through an interviewer‐administered questionnaire.

For the analysis of FBS, 10 hours of overnight fast was considered, and for triglycerides and HDL cholesterol, 12 hours of overnight fast was considered. FBS analysis was done using Biorex diagnostics, glucose kit where FBS <100 mg/dL was considered as normal. For lipid profile, separated serum was analyzed using Stanbio cholesterol LiquiColor kit, where TG < 150 mg/dL, HDL > 40 mg/dL (for males) and > 50 mg/dL (for female) were considered as normal. WC < 90 cm for South Asian males and <80 cm for South Asian females were considered normal.^13^, ^14^ Blood pressure was measured using a mercury sphygmomanometer by a qualified medical professional following 10 to 15 minutes resting period while the subject was in the seated position. WC was measured according to the standard method using a validated non‐stretchable commercial tape. Subjects were in the standing position with arms at sides of the body and feet positioned close together, in a relaxed posture, breathing naturally and without contracting any abdominal muscles. When the subject places hands on hip bones, it was considered to be at the approximate midpoint (at the level of the belly button) between the lower margin of the last palpable rib and the top of the iliac crest. The measurement was taken at that point by circling the tape parallel to the floor around the body to the starting point. Percentages of MetS among hypertensive and non‐hypertensive were determined according to both the modified National Cholesterol Education Program (NCEP) adult treatment panel ΙΙΙ (ATP ΙΙΙ) and new IDF criteria (2005). According to the new IDF criteria, MetS is defined as the presence of the following:

1. Large waistline (central obesity): WC ≥90 cm for South Asian male and ≥ 80 cm for South Asian female, plus any two of the following four features:
2. High triglyceride level: ≥150 mg/dL (1.7 mmol/L), or obtaining treatment for high TG
3. Decreased high density lipoprotein cholesterol: <40 mg/dL for male, <50 for female, or obtaining treatment for low HDL cholesterol.
4. Raised blood pressure: systolic BP ≥ 130 or diastolic BP ≥ 85 mm Hg, or obtaining treatment for hypertension.
5. Increased fasting blood sugar (glucose) level: ≥100 mg/dL (5.6 mmol/L) or obtaining treatment for high blood sugar

According to modified ATP ΙΙΙ (2005) criteria, MetS is defined as the presence of at least three of the above‐mentioned five features. All five components were taken into account in this study.^15^, ^16^

### 2.2 Statistical analysis

Data were analyzed using statistical package for social sciences (SPSS) version 21. Significance of mean differences of MetS related characteristics between hypertensive and non‐hypertensive groups was done using independent sample *t*‐test. For Means ± SD and frequencies, descriptive analysis was done. Fisher's exact test (two‐tailed) was used to determine the significance of association between two categorical variables (continuous variables were categorized according to cutoff values). *P* value was used to measure the significance. If *P* < .05, the observed difference or association was considered as significant.

## 3 RESULTS

The mean age of total, male, and female subjects were 47.6±6.2, 47.03±6.4, and 48.35±6.15 years, respectively. The mean age of hypertensive subjects (50.13±4.9 years) was significantly higher (*P* < .001) than non‐hypertensives (45.25±6.6 years). Medical information and lifestyle information of the study subjects are presented in Table 1. There were no significant differences encountered with regard to the physical inactivity, diet control, occupation, familial hypertension, familial diabetes, familial heart disease, and familial dyslipidemia between hypertensive and non‐hypertensive subjects.

**TABLE 1:** Medical information and lifestyle information of the study subjects

Characteristics	Percentage of subjects among hypertensives (%) (n = 60)	Percentage of subjects among non‐hypertensives (%) (n = 60)	*P* value
Diet control	40	33	.57
Occupation	83	72	.19
Physical activity >2 to 3 h/wk	52	55	.86
Familial hypertension	42	27	.12
Familial DM	43	33	.35
Familial heart disease	10	17	.42
Familial dyslipidemia	12	17	.60

Mean values of assessed biochemical parameters are presented in Table 2. both hypertensive males and females had a higher mean FBS compared with non‐hypertensive subjects where a significantly (*P* < .001) higher FBS was observed only in male hypertensive subjects compared with non‐hypertensive males. Mean TG was significantly (*P* = .005) higher in hypertensive females compared with non‐hypertensive females. The mean WC was higher than the normal reference level in both hypertensive females and non‐hypertensive females, but the value was significantly (*P* = .002) higher in hypertensive males compared with non‐hypertensive males.

**TABLE 2:** Mean values of biochemical parameters between hypertensive and non‐hypertensive groups

	Hypertensive males Mean ± SDF	Non‐hypertensive males Mean ± SD	*P* value	Hypertensivefemales Mean ± SD	Non‐hypertensive females Mean ± SD	*P* value
FBS (mg/dL)	101.2 ± 26.9*	79.5 ± 15.4	<.001	114.6 ± 69.0	86.6 ± 9.1	.38
TG (mg/dL)	126.2 ± 106.2	132.3 ± 64.3	.79	123.6 ± 55.5*	87.6 ± 36.9	.005
HDL (mg/dL)	40.4 ± 9.3	38.5 ± 7.7	.39	47.4 ± 10.1	46.2 ± 7.3	.60
WC (cm)	95.1 ± 8.7*	88.1 ± 7.7	.002	90.6 ± 8.1	86.5 ± 9.0	.08

* Difference is significant at .05 level.

The presence of MetS in this study population was assessed using modified ATP III and new IDF criteria, and the data are given in Table 3. According to new IDF criteria, 38 subjects (63%) of the hypertensive group and 12 subjects (20%) in non‐hypertensive had MetS. According to modified ATPIII criteria, 41 subjects (68%) in the hypertensive group and 12 (20%) subjects in non‐hypertensive group presented with MetS. The percentage of subjects with MetS was significantly (*P* < .001) higher in the hypertensive group compared with the non‐hypertensive group according to both modified ATP III and new IDF criteria.

**TABLE 3:** Percentage of subjects with metabolic syndrome in hypertensive and non‐hypertensive groups

	MetS in hypertensives (%)	MetS in non‐hypertensives (%)	*P* value
Modified ATP III (2005) criteria			
Total (n = 60 in each group)	68	20	.000**
Male (n = 30 in each group)	60	17	.001**
Female (n = 30 in each group)	77	23	.000**
New IDF (2005) criteria			
Total (n = 60 in each group)	63	20	.000**
Male (n = 30 in each group)	53	17	.006**
Female (n = 30 in each group)	73	23	.000**

** Difference is significant at .01 level.

The percentage of subjects with FBS, TG, HDL, and WC beyond the risk cutoff values are given in Table 4. The percentage of subjects with increased FBS was significantly (*P* = .025) higher in the total hypertensive group and hypertensive males compared with respective non‐hypertensive groups (Table 4). The majority of the females in hypertensive (90%) and non‐hypertensive groups (77%) had increased WC. The percentage of subjects with decreased HDL than normal reference level was significantly (*P* = .002) higher in hypertensive males than non‐hypertensive males, while a significant difference between female hypertensives and non‐hypertensives was not observed. The percentage of subjects with increased FBS, WC, TG were significantly higher in MetS subjects than non‐MetS individuals.

**TABLE 4:** Percentage of subjects with increased FBS, TG, and WC than the normal reference and decreased HDL than the normal reference

Biochemical test	Hypertension n = 60 (%)	Non‐hypertension n = 60 (%)	*P* value	MetS according to modified ATP III n = 53 (%)	Non‐MetS, n = 67 (%)	*P* value
Increased FBS (≥100 mg/dL)						
Total	38.3	8.30	.03*	52.8	8.90	.000**
Male	40.0	6.60	.005**	47.8	8.10	.001**
Female	36.6	30.0	.79	56.7	10.0	.001**
Increased TG (≥150 mg/dL)						
Total	28.3	21.6	.53	43.3	10.4	.000**
Male	26.6	33.3	.78	52.2	16.2	.004**
Female	30.0	10.0	.10	36.7	3.30	.002**
Decreased HDL						
Total	58.3	70.0	.25	81.1	50.7	.001**
Male <40 mg/dL	60.0	63.3	>.99	86.9	45.9	.002**
Female <50 mg/dL	56.6	76.6	.17	76.7	56.7	.17
Increased WC						
Total	80.0	58.3	.02*	96.2	47.7	.000**
Male ≥90 cm	70.0	40.0	.04*	95.6	29.7	.000**
Female ≥80 cm	90.0	76.6	.30	96.7	70.0	.012*

*Note*: Any three of the following (increase in blood pressure, FBS, WC, TG, and decrease in HDL) were considered as subjects with MetS.

* Difference is significant at .05 level

** Difference is significant at .01 level.

## 4 DISCUSSION

MetS is a health burden that has become a challenging problem worldwide. This study aimed to identify whether a normal routine measurement of blood pressure would pave way to detect development of MetS early. Supporting the hypothesis, the key finding of the present study was the significantly higher percentage of subjects with MetS in patients with hypertension compared with their non‐hypertensive counterparts according to both methods of diagnosis of MetS (modified ATP III and new IDF criteria). Further, the MetS subjects with hypertension presented with raised FBS and WC. A study done in Brazil has observed the prevalence of MetS in hypertensives as 71.6% and 82.4% according to ATPIII (2001) and IDF (2005) criteria, respectively.^17^ The increase in FBS and WC observed in hypertensive subjects in the present study is supported by another study carried out by De Silva ST et al in 2014.^9^

Supporting the findings of the present study, a study conducted in Europe has reported that 66.5% of uncontrolled hypertensive patients had MetS and 35.5% of patients with controlled blood pressure had MetS.^18^ In accordance with our study, other studies have observed an association between hypertension and diabetes, where people with hypertension appeared to have an increased risk for developing diabetes.^19^, ^20^ Further, in the present study, significantly higher FBS level was observed in hypertensive males and a higher percentage of hypertensive subjects had elevated FBS. Several studies have found a close association between obesity and hypertension.^21^, ^22^ Central obesity shows a strong association with hypertension compared with other parameters that assess obesity.^23^, ^24^

Central obesity, which is depicted by increased WC, is considered as one of the main underlying factors and one of the main features of MetS.^13^ Hence, central obesity can be used as an early predictor of MetS. In the present study, WC was used to measure central obesity. WC was significantly higher in the male hypertensive group, while mean WC was higher than the risk cutoff value in both hypertensive and non‐hypertensive females. Increased WC in these normotensive subjects is alarming and suggests the need for routine monitoring to prevent the onset of MetS with time.

According to a report of ATP III, MetS is associated with elevated triglycerides.^25^ Although only female hypertensive group had a significantly higher mean triglyceride level, the increased TG and decreased HDL observed in non‐hypertensive subjects indicate the need for random screening and education about the subsequent effects of the altered lipid profile and anthropometric effects that may lead to onset of MetS. The major drawback of our study was limiting the study setting to one location, which is categorized as an urban area in Sri Lanka. Data collection was done by four researchers, hence bias in reporting is a possibility. Inability to recall all facts by the participants with regard to lifestyle and medical information was an additional drawback.

In summary, the percentage of subjects with increased FBS, WC, and MetS was significantly higher in the hypertensive group compared with the non‐hypertensive group.

Hence, the findings of the present study not only indicate that hypertensive subjects are more prone to have MetS but also emphasize the urgent need to develop national strategies for the early detection and preventive measures to make people aware of the metabolic syndrome.

## CONFLICT OF INTEREST

The authors declare that they have no conflict of interests.

## AUTHOR CONTRIBUTIONS

Conceptualization: Usha Hettiaratchi, Lohini Athiththan

Data Curation: Roshali Senarathne, Usha Hettiaratchi, Lohini Athiththan

Formal Analysis: Roshali Senarathne

Funding Acquisition: Lohini Athiththan

Investigation: Roshali Senarathne, Nirodha Dissanayake, Riyaza Hafiz, Sumara Zaleem

Methodology: Usha Hettiaratchi, Lohini Athiththan, Roshali Senarathne, Nirodha Dissanayake

Recourses: Usha Hettiaratchi, Lohini Athiththan

Software: Roshali Senarathne, Lohini Athiththan

Supervision: Usha Hettiaratchi, Lohini Athiththan

Validation: Usha Hettiaratchi, Lohini Athiththan

Writing—Original Draft: Roshali Senarathne

Writing—Review and Editing: Usha Hettiaratchi, Lohini Athiththan

All authors have read and approved the final version of the manuscript.

Lohini Athiththan had full access to all of the data in this study and takes complete responsibility for the integrity of the data and the accuracy of the data analysis.

## TRANSPARENCY STATEMENT

Lohini Athiththan affirms that this manuscript is an honest, accurate, and transparent account of the study that is being reported; and no important aspects of the study have been omitted; and that any discrepancies from the study as planned (and, if relevant, registered) have been explained.

## ETHICS STATEMENT

Approval for the present work was obtained from the Ethics review committee of Faculty of Medical Sciences, University of Sri Lanka, Sri Lanka (reference numbers‐ MLS 06/2015, B Pharm 05/2015).

## Data Availability

The authors confirm that all data supporting the findings of this study are available within the article [and/or] its supplementary materials.
